# Comprehensive HIV/AIDS knowledge among Rwandan men aged 50–59: evidence from the 2019–20 RDHS

**DOI:** 10.1186/s12981-025-00825-6

**Published:** 2025-12-18

**Authors:** Jessy Rugeyo, Joseph Kawuki

**Affiliations:** 1 Department of Sociology and Demography, The College for Health, Community and Policy (HCAP), University of Texas at San Antonio, San Antonio, TX USA; 2 Program in Public Health, Department of Family, Population, & Preventive Medicine, Stony Brook University, Stony Brook, NY USA

**Keywords:** HIV/AIDS knowledge, Aging, Rwanda, HIV prevention, Education, Men’s health

## Abstract

**Background:**

Comprehensive HIV/AIDS knowledge is essential for prevention, yet older adults-particularly men aged 50–59 years-remain underrepresented in HIV research and programming across sub-Saharan Africa. Despite Rwanda’s strong HIV response, limited evidence exists on HIV knowledge among older men. This study assessed the prevalence and determinants of comprehensive HIV/AIDS knowledge among Rwandan men aged 50–59 years. The analysis was guided by a conceptual framework incorporating predisposing, enabling, and contextual factors that shape HIV knowledge among older men.

**Methods:**

This cross-sectional study used data from the 2019–20 Rwanda Demographic and Health Survey (RDHS), analyzing a weighted sample of 665 men aged 50–59 years. Comprehensive HIV knowledge was defined using six standard DHS indicators. Weighted descriptive statistics, bivariable analyses, and multivariable logistic regression were conducted to identify associated factors, adjusting for the survey design. Results are presented as adjusted odds ratios (AORs) with 95% confidence intervals (CIs).

**Results:**

Overall, 69.9% (95% CI: 66.2–73.4%) of men aged 50–59 years demonstrated comprehensive HIV/AIDS knowledge. In adjusted analyses, having secondary education (AOR = 3.18; 95% CI: 1.31–7.75) and health insurance coverage (AOR = 1.71; 95% CI: 1.07–2.73) were significantly associated with higher odds of comprehensive knowledge. Other factors, including mobile phone ownership and internet use, were not significant after adjustment.

**Conclusions:**

Comprehensive HIV knowledge among Rwandan men aged 50–59 years remains below global prevention targets. Education and health insurance coverage were the most important enabling factors, consistent with the study’s conceptual framework. Integrating HIV education into aging-friendly and insurance-linked health services may strengthen awareness and support Rwanda’s continued progress toward HIV prevention goals.

## Introduction

Acquired Immune Deficiency Syndrome (AIDS), caused by the Human Immunodeficiency Virus (HIV), remains a critical global public health challenge despite significant progress in reducing AIDS-related mortality. By the end of 2023, an estimated 39.9 million people worldwide were living with HIV [1]. The burden is disproportionately high in the World Health Organization (WHO) African Region, where approximately 3.4% of adults—or one in every thirty—are HIV-positive, accounting for over two-thirds of the global HIV-positive population [2]. In sub-Saharan Africa, men represent about 44% of people living with HIV, yet they remain an understudied demographic in HIV research. AIDS continues to be a leading cause of death among African adult men [3, 4], and projections indicate a rising trend in HIV prevalence among older populations, with an estimated 6 to 10 million individuals aged 50 years and older living with HIV in sub-Saharan Africa in the coming decades [5].

Global efforts to combat HIV/AIDS have led to significant advancements, including the widespread scale-up of antiretroviral therapy (ART), which has substantially reduced morbidity and mortality. In 2016, the United Nations Political Declaration on HIV/AIDS set an ambitious target to end the AIDS epidemic by 2030 [5, 6]. Central to this goal is the 95-95-95 strategy, which aims to ensure that 95% of people living with HIV know their status, 95% of those diagnosed receive sustained ART, and 95% of those on treatment achieve viral suppression. However, persistent gaps in HIV knowledge, testing and treatment initiation mean most countries are unlikely to meet this target [6].

Rwanda has made remarkable strides in HIV prevention and treatment through accelerated testing, immediate ART initiation (“test and treat”), and evidence-based interventions such as condom distribution, targeted testing, prevention of mother-to-child transmission (PMTCT), voluntary medical male circumcision (VMMC), and community education programs [7, 8]. Despite these efforts, low awareness of HIV knowledge and self-testing among Rwandan men remains a critical barrier to testing uptake [9].

Comprehensive HIV/AIDS knowledge is a crucial determinant of prevention behaviors, as it equips individuals with accurate information on transmission, risk reduction, and treatment, while dispelling harmful misconceptions [10, 11]. However, studies across sub-Saharan Africa reveal low levels of comprehensive HIV/AIDS knowledge, ranging from 19.3% in Ethiopia to 59% in Ghana [12–16]. Factors such as socioeconomic status, education, media exposure, and geographic location influence HIV knowledge levels [15, 17]. Despite extensive HIV programming in Rwanda, there is a striking lack of empirical data on HIV knowledge among older men. National surveys often aggregate adults into a single 15–49 age group or focus primarily on women and key populations, leaving men aged 50 years and older underrepresented in the evidence base [12, 18]. Existing studies on older adults are typically clinic-based or rely on small convenience samples, limiting generalizability and overlooking structural determinants such as media access and healthcare proximity [19]. This knowledge gap may contribute to delayed HIV diagnosis and treatment initiation among older men in the region [20].

To address this knowledge gap, this study aimed to estimate the prevalence of comprehensive HIV/AIDS knowledge and identify its associated factors among Rwandan men aged 50–59 years using data from the 2019-20 Rwanda Demographic and Health Survey (RDHS). Guided by a conceptual framework incorporating predisposing, enabling, and contextual factors, the study sought to identify the determinants shaping HIV knowledge within this understudied demographic. Findings from this analysis can inform targeted interventions that integrate HIV education into aging-friendly and insurance-linked health services, thereby improving awareness, prevention behaviors such as testing and condom use, and contributing to Rwanda’s progress toward ending the HIV epidemic.

## Conceptual framework

Our hypothesis is that men aged 50–59 achieve comprehensive HIV knowledge through three interconnected pathways:

(i) Predisposing factors—such as age, education, and religion—which influence baseline health literacy and prevailing social norms.
(ii) Enabling factors—including health insurance coverage, household wealth, and exposure to media—that represent access to information and interaction with the health system; and.
(iii) Contextual factors—such as rural or urban residence and province or region—that capture structural and programmatic differences in information environments.

These categories guided the a priori selection of covariates for the multivariable analysis.

## Methods

### Data source and sampling

This study utilized data from the 2019–20 Rwanda Demographic and Health Survey (RDHS), a nationally representative cross-sectional survey. Specifically, we analyzed the men’s recode file, which contains individual-level responses from male participants. The RDHS is part of the broader Demographic and Health Surveys (DHS) Program, conducted in over 85 low- and middle-income countries to monitor key health and demographic indicators, including fertility, family planning, immunization, maternal and child health, nutrition, and HIV/AIDS [21]. The RDHS employed a two-stage stratified sampling design to ensure national representativeness. In the first stage, clusters (typically villages or urban neighborhoods) were selected with probability proportional to size, and in the second stage, households within each cluster were randomly sampled. Detailed sampling procedures are available in the DHS final report [22]. From the total sample of 6513 men aged 15–59 years who consented and participated in the survey, our analysis focused on men aged 50–59 years. Of the 679 men aged 50–59 years identified in the RDHS men’s dataset, 665 had complete data on all six HIV knowledge indicators and were therefore included in the final analytical sample. This minor difference reflects a combination of missing responses and the application of sampling weights to produce nationally representative estimates. The dataset is publicly available for download from the DHS Program website.

### Study variables and measurements

#### Dependent variable

The primary outcome of this study was comprehensive knowledge about HIV/AIDS, operationalized according to the standard DHS definition. This variable represents a latent construct derived from six binary (yes/no) indicators assessing respondents’ accurate understanding of HIV transmission and prevention. Participants were evaluated based on their responses to the following questions:

1. Can a healthy-looking person have HIV? (Correct: Yes)
2. Can HIV be transmitted through mosquito bites? (Correct: No)
3. Does consistent condom use during sex reduce HIV risk? (Correct: Yes)
4. Does having one mutually faithful, uninfected sexual partner reduce HIV risk? (Correct: Yes)
5. Can HIV be transmitted through witchcraft or supernatural means? (Correct: No)
6. Can HIV be transmitted by sharing food with a person living with AIDS? (Correct: No)

A respondent was classified as having comprehensive HIV knowledge if they answered all six questions correctly-that is, “Yes” for items 1, 3, and 4, and “No” for items 2, 5, and 6 [23, 24].

Sensitivity analyses were conducted using two alternative scoring approaches because this definition is relatively strict and may underestimate partial knowledge: (i) defining comprehensive knowledge as correctly answering at least five of six questions (≥ 5/6 threshold), and (ii) creating a continuous 0–6 knowledge score, where higher values indicate greater HIV knowledge. Results from both alternate measures were comparable to those from the core binary outcome in terms of both direction and magnitude.

#### Explanatory variables

Fourteen explanatory variables were examined, selected a priori based on theoretical relevance and prior empirical evidence linking sociodemographic and contextual factors to HIV knowledge [14, 15, 23, 25]. Guided by a conceptual framework distinguishing predisposing, enabling, and contextual factors, the variables were grouped into two levels: individual-level and community-level determinants. At the individual level, predisposing factors included age, marital status, religious affiliation, and educational attainment. Age was treated as a continuous variable. Marital status was categorized as never married, married/in union, or widowed/divorced/separated. Religious affiliation was classified as Christian, Muslim, or other. Educational attainment was grouped into no education, primary, secondary, and higher education categories. Enabling factors captured access to information and health services. These included employment status (yes/no), HIV testing history (yes/no), and health insurance coverage (yes/no). Media exposure was measured through three binary indicators reflecting whether respondents reported regularly listening to the radio, watching television, or reading newspapers. In addition, digital access was assessed through two variables: internet use (yes/no) and mobile phone ownership (yes/no).

At the community level, contextual factors were incorporated to account for structural and geographic influences on knowledge. The household wealth index, representing relative socioeconomic status, was divided into five quintiles (poorest, poorer, middle, richer, and richest). Geographic factors included region of residence (Kigali, South, West, North, East) and type of residence (urban or rural). All categorical variables were treated as nominal in the analysis. This theoretically informed selection of explanatory variables allowed for a comprehensive assessment of the individual and contextual determinants associated with comprehensive HIV knowledge among men aged 50–59 in Rwanda.

#### Covariate selection

Following the previously outlined conceptual framework, we incorporated variables that represented contextual (residence, province/region), enabling (health insurance, affluence, media exposure), and predisposing (age, education, and religion) factors. These factors were not chosen on the basis of statistical significance, but were kept a priori to represent likely routes to HIV knowledge.

### Data management and statistical analysis

To ensure nationally representative estimates, sampling weights (mv005/1,000,000) were applied to adjust for unequal probabilities of selection across strata and clusters in the 2019–20 Rwanda Demographic and Health Survey (RDHS) [26]. All analyses were performed using IBM SPSS Statistics version 25.0 with the Complex Samples module, which correctly accounts for the RDHS’s stratified, multi-stage cluster design [27]. The design variables for primary sampling units (clusters) and strata were specified to ensure accurate variance estimation. The analysis proceeded in three stages. First, descriptive statistics were computed for all variables. Categorical variables were summarized as weighted frequencies and percentages. Second, bivariate relationships between comprehensive HIV knowledge and each explanatory variable were assessed using Rao–Scott chi-square tests, which adjust for complex survey design effects, with statistical significance set at *p* < 0.05.

Third, binary logistic regression was used to identify independent predictors of comprehensive HIV knowledge. Unadjusted models produced crude odds ratios (COR) with 95% confidence intervals (CI). Variables significant at *p* < 0.05 in bivariate analyses, as well as those selected on theoretical grounds (education, media exposure, insurance coverage, and wealth index), were included in the multivariable model. A backward elimination procedure was employed to achieve a parsimonious model while retaining theoretically relevant covariates. The final adjusted model generated adjusted odds ratios (AOR) with 95% CIs, controlling for potential confounders.

Model adequacy was evaluated using the Hosmer–Lemeshow goodness-of-fit test (*p* >0.05 indicating adequate fit) [28], and multicollinearity was examined using variance inflation factors (VIF), all of which were below 10. To assess sample adequacy, the events-per-variable (EPV) heuristic was applied. With a 69.9% prevalence of comprehensive knowledge (≈ 465 events and ≈ 200 non-events) and 14 predictors, EPV values were approximately 33 based on events and 14 based on the smaller outcome cell, both above conventional thresholds. Precision was conveyed through 95% confidence intervals rather than post-hoc power estimates [29].

To evaluate the robustness of the main outcome definition, sensitivity analyses were conducted using two alternative measures of HIV knowledge: (i) a threshold of ≥ 5 correct responses out of six and (ii) a continuous 0–6 knowledge score. Both approaches yielded directionally consistent and statistically comparable results with the main binary outcome.

## Results

### Socio-demographic and economic characteristics of the study participants

A total of 665 men aged 50–59 from the 2019–20 Rwanda Demographic Health Survey were included in the analysis. Most participants (93.0%) were married or in union, while 4.9% were widowed, divorced, or separated. A significant proportion (65.5%) had attained primary education, and 24.4% had no formal education. Regarding employment status, the majority of participants (96.0%) were currently working. Most men identified as Christian (95.2%), with Muslims comprising 2.9% of the sample. Over two-thirds (66.3%) were previously tested for HIV. A large proportion (84.2%) reported having health insurance coverage.

Media exposure varied among participants, with 87.6% regularly listening to the radio, 55.4% watching television, and 22.4% reading newspapers. Internet usage was low at 7.3%, while mobile phone ownership was comparatively higher (56.9%). In terms of wealth distribution, approximately equal proportions were observed across all quintiles, with the poorest and richest quintiles accounting for 14.2% and 19.3% respectively. Geographically, participants were evenly distributed across Rwanda’s regions, with the highest proportion from the East (25.5%) and the lowest from Kigali and the North (14.0% each). The majority resided in rural areas (83.5%) as shown in Table 1.

### Prevalence of comprehensive knowledge about HIV/AIDS

The overall prevalence of comprehensive HIV/AIDS knowledge among men aged 50–59 was 69.9% (95% CI: 66.2–73.4%). Individually, most participants correctly identified that a healthy-looking person could have HIV (93.6%), consistent condom use during sex reduces HIV transmission (93.3%), and having one mutually faithful uninfected partner reduces HIV risk (91.1%). However, misconceptions persisted, with 11.3% believing HIV could be transmitted through mosquito bites, 4.7% through sharing food with an HIV-positive individual, and 1.8% through witchcraft or supernatural means, as detailed in Table 2.

**Table 1:** Weighted sociodemographic characteristics of men aged 50–59, Rwanda DHS 2019–20

Variable	Total weighted frequency (%)	Comprehensive HIV/AIDS knowledge		*p*-value**
		No (%)	Yes (%)	
Comprehensive HIV/AIDS Knowledge		200 (30.10)	465 (69.90)	
Individual level variables				
*Marital status*				
Never married	14 (2.10)	4 (33.90)	9 (66.10)	0.94
Married / in union	620 (93.00)	186 (29.90)	434 (70.10)	
Widowed / divorced / separated	33 (4.90)	10 (31.50)	22 (68.50)	
*Religious affiliation*				
Christian	635 (95.20)	193 (30.50)	441 (69.50)	0.13
Muslim	20 (2.90)	2 (10.80)	17 (89.20)	
Other	13 (1.90)	5 (41.70)	7 (58.30)	
*Currently working*				
No	27 (4.00)	3 (12.30)	24 (87.70)	0.05
Yes	640 (96.00)	197 (30.80)	442 (69.20)	
*Educational level*				
No education	163 (24.40)	56 (34.90)	105 (65.10)	**0.02**
Primary	437 (65.50)	134 (30.70)	302 (69.30)	
Secondary	44 (6.60)	6 (13.60)	38 (86.40)	
Higher	24 (3.60)	4 (16.60)	20 (83.40)	
*Ever tested for HIV*				
No	225 (33.70)	61 (27.20)	163 (72.80)	0.28
Yes	442 (66.30)	140 (31.50)	303 (68.50)	
*Listen to radio*				
No	83 (12.40)	21 (26.00)	61 (74.00)	0.39
Yes	584 (87.60)	179 (30.70)	405 (69.30)	
*Watch television*				
No	297 (44.60)	85 (28.80)	211 (71.20)	0.51
Yes	370 (55.40)	115 (31.10)	255 (68.90)	
*Read newspaper*				
No	517 (77.60)	159 (30.80)	357 (69.20)	0.49
Yes	150 (22.40)	41 (27.70)	108 (72.30)	
*Use Internet*				
No	618 (92.70)	192 (31.20)	424 (68.80)	**0.02**
Yes	49 (7.30)	8 (16.30)	41 (83.70)	
*Covered by health insurance*				
No	106 (15.80)	43 (40.80)	63 (59.20)	**0.02**
Yes	561 (84.20)	157 (28.10)	403 (71.90)	
*Wealth index*				
Poorest	95 (14.20)	38 (39.90)	57 (60.10)	0.08
Poorer	147 (22.00)	48 (32.90)	98 (67.10)	
Middle	150 (22.50)	43 (28.80)	107 (71.20)	
Richer	146 (21.90)	43 (29.20)	103 (70.80)	
Richest	129 (19.30)	29 (22.30)	100 (77.70)	
*Owns mobile phone*				
No	287 (43.10)	102 (35.50)	185 (64.50)	**0.01**
Yes	380 (56.90)	99 (26.00)	281 (74.00)	
*Region*				
Kigali	94 (14.00)	21 (22.00)	73 (78.00)	0.11
South	159 (23.80)	54 (33.80)	105 (66.20)	
West	150 (22.50)	40 (26.30)	111 (73.70)	
North	94 (14.10)	35 (37.50)	58 (62.50)	
East	170 (22.50)	52 (30.50)	118 (69.50)	
*Residence*				
Urban	110 (16.50)	25 (22.80)	85 (77.20)	**0.04**
Rural	557 (83.50)	175 (31.50)	380 (68.50)	

Bolded values indicate statistically significant associations at *p* < 0.05 based on Rao–Scott chi-square tests

**Table 2:** Comprehensive HIV/AIDS knowledge among men aged 50–59, Rwanda DHS 2019–20 (weighted *N* = 665)

Comprehensive knowledge question	Yes (%)	No (%)
Always using condoms during sex can reduce the risk of getting HIV	632 (93.3)	47 (6.7)
Having one sex partner only, who has no other partners, can reduce the risk of getting HIV	616 (91.1)	63 (8.9)
A health-looking person can have HIV	633 (93.6)	46 (6.4)
Can get HIV by witchcraft or supernatural means	12 (1.8)	667 (98.2)
Can get HIV by sharing food with a person who has AIDS	34 (4.7)	645 (95.3)
Can get HIV from mosquito bites	79 (11.3)	600 (88.7)
Comprehensive knowledge of HIV/AIDS (Total)	468 (69.9)	211 (30.1)

Percentages and frequencies are weighted to ensure national representativeness. Totals may not sum to 665 due to weighting adjustments

### Factors associated with comprehensive knowledge of HIV/AIDS

Bivariable logistic regression initially identified several factors associated with comprehensive HIV/AIDS knowledge among older Rwandan men, including educational attainment, internet usage, health insurance coverage, mobile phone ownership, and place of residence. However, after controlling for potential confounders in the multivariable logistic regression analysis (Table 3), only educational attainment and health insurance coverage remained statistically significant predictors. Specifically, men with secondary education demonstrated more than threefold higher odds (AOR = 3.18; 95% CI: 1.31–7.75) of having comprehensive HIV/AIDS knowledge compared to those without formal education. Similarly, health insurance coverage was significantly associated with higher odds of comprehensive HIV/AIDS knowledge (AOR = 1.71; 95% CI: 1.07–2.73). Other variables examined in the adjusted model, such as mobile phone ownership, internet usage, and urban residence, did not retain statistical significance. Sensitivity analyses using alternative definitions of HIV knowledge (≥ 5 of 6 correct responses and a continuous 0–6 score) produced results consistent in both direction and magnitude with the main binary outcome, confirming the robustness and stability of the findings.

## Discussion

Our study found that 69.9% of Rwandan men aged 50–59 demonstrated comprehensive HIV/AIDS knowledge—a proportion notably higher than estimates from comparable older-adult populations in other sub-Saharan African countries. For instance, in South Africa and Lesotho, only 36% of adults aged 50 and above attained adequate HIV-related knowledge, attitudes, and practices (KAP) scores at baseline, with men exhibiting significantly lower odds of sufficient knowledge than women (female vs. male AOR = 1.6) [30]. Similarly, in peri-urban Ghana, older adults 50 years and older displayed predominantly “average” knowledge levels—62% for transmission, 58% for prevention, and 60% for signs/symptoms—though male-specific data were unavailable [20]. Evidence from Uganda further highlights gender disparities disadvantaging older men: among adults aged 45–59, men were less likely to be aware of their HIV status than women (female vs. male AOR = 1.26), with lower comprehensive knowledge correlating with poorer status awareness [31]. Additionally, in rural Ugandan districts, only 53% of individuals aged 50 and older had undergone recent HIV testing, though correct knowledge on key items was positively associated with testing uptake [32]. Collectively, these regional comparisons suggest that older men in sub-Saharan Africa often exhibit suboptimal HIV knowledge and engagement relative to public health targets. While Rwanda’s estimate is comparatively favorable, it still falls below the UNAIDS global benchmark of 95% [16]. This relatively higher performance may be attributed to Rwanda’s robust healthcare infrastructure, effective national HIV/AIDS awareness campaigns, and sustained community-based education initiatives. In addition, Rwanda has made substantial investments in strengthening its health and education systems following the 1994 genocide against the Tutsi, potentially contributing to the more favorable knowledge outcomes observed [33].

When benchmarked against younger men, Demographic and Health Survey (DHS)-based estimates reveal that comprehensive HIV knowledge remains suboptimal among males aged 15–24, with only 39% in Ethiopia [24], 45% in Uganda [34], and 22% in Nigeria [35] demonstrating adequate understanding. These figures fall substantially below the UNAIDS 95% target and, in several contexts, are either comparable to or lower than those of their female peers, highlighting persistent knowledge disparities among young men across the region. A broader multi-country analysis further corroborates this trend, indicating that only approximately half of adult men in sub-Saharan Africa possess comprehensive HIV knowledge [4]. Notably, cross-country variations in HIV/AIDS knowledge prevalence may reflect methodological differences in measurement tools. For example, Fenny et al. [14] assessed comprehensive knowledge using four indicators in Ghana, whereas Ochako et al. [25] in Kenya and Estifanos et al. [15] in Uganda employed five. In contrast, the present study utilized six standardized DHS indicators, providing a more robust and comprehensive assessment. Additionally, many comparative studies have primarily focused on women of reproductive age-a demographic with distinct health-seeking behaviors and greater exposure to targeted interventions compared to older men. These disparities in study populations and measurement approaches constrain direct comparability but nevertheless stress the persistent gaps in HIV knowledge across diverse demographic groups.

The conceptual framework that guided this study is consistent with these findings. It posited that comprehensive HIV knowledge among men aged 50–59 would be influenced by three categories of factors: predisposing (e.g., education, religion), enabling (e.g., health insurance, household wealth, media exposure), and contextual (e.g., rural/urban residence, province/region). Consistent with this model, enabling factors-particularly education, health-insurance coverage, and media exposure-showed the strongest associations with comprehensive HIV knowledge, suggesting that access to information and engagement with the health system may be more important determinants than baseline demographic characteristics.

Despite the relatively higher prevalence reported in our study, the observed level of comprehensive HIV/AIDS knowledge among older Rwandan men remains suboptimal, posing significant public health concerns. As the population ages, older adults become increasingly susceptible to delayed HIV diagnosis and poorer treatment outcomes [19]. Therefore, targeted HIV education strategies tailored to this demographic are urgently needed. Integrating comprehensive HIV education into the routine geriatric health services, alongside expanding community outreach and awareness campaigns, could substantially enhance knowledge, reduce stigma, and promote preventive behaviors aligned with international HIV response goals.

Educational attainment emerged as a consistently significant predictor of comprehensive HIV knowledge in our study. Specifically, men with secondary education were over three times more likely to possess accurate HIV knowledge compared to those without formal education. This finding aligns with prior evidence from Ghana and Kenya, where higher educational attainment was associated with increased awareness of HIV prevention methods [14, 25]. Education plays a central role not only in enhancing literacy but also in equipping individuals with the skills necessary to access, interpret, and apply health-related information. In Rwanda, initiatives under the National Program for HIV/AIDS Control (PNLS) have long promoted school-based and community-level education programs, stressing the value of expanding quality education to mitigate HIV risk among older populations.

**Table 3:** Factors associated with comprehensive HIV/AIDS knowledge among men aged 50–59: Rwanda DHS 2019–20

Variable	Crude odds ratio, COR (95% CI)	*p*-value*	Adjusted odds ratio, AOR (95% CI)	*p*-value**
*Marital status*		0.93		0.13
Never married	1		1	
Married / in union	1.20 (0.40–3.64)		1.61 (0.45–5.77)	
Widowed / divorced / separated	1.11 (0.28–4.49)		6.25 (0.95–40.89)	
*Religious affiliation*		0.15		0.10
Christian	1		1	
Muslim	3.62 (0.88–14.95)		4.46 (0.93–21.28)	
Other	0.61 (0.18–2.07)		0.54 (0.16–1.89)	
*Currently working*		0.07		0.11
No	1		1	
Yes	0.32 (0.09–1.09)		0.33 (0.09–1.28)	
*Educational level*		**0.02**		**0.02**
No education	1		1	
Primary	1.21 (0.82–1.79)		1.19 (0.80–1.77)	
Secondary	3.40 (1.41–8.20)		**3.18 (1.31–7.75)**	
Higher	2.68 (0.93–7.71)		2.77 (0.95–8.03)	
*Ever tested for HIV*		0.28		0.66
No	1		1	
Yes	0.81 (0.56–1.18)		0.70 (0.48–1.03)	
*Listen to radio*		0.39		0.72
No	1		1	
Yes	0.80 (0.47–1.34)		0.59 (0.33–1.05)	
*Watch television*		0.51		0.33
No	1		1	
Yes	0.89 (0.64–1.25)		0.83 (0.57–1.20)	
*Read newspaper*		0.49		0.70
No	1		1	
Yes	1.16 (0.76–1.77)		0.92 (0.59–1.43)	
*Use Internet*		**0.02**		0.72
No	1		1	
Yes	2.33 (1.16–4.70)		1.24 (0.40–3.83)	
*Covered by health insurance*		**0.02**		**0.03**
No	1		1	
Yes	1.77 (1.11–2.81)		1.71 (1.07–2.73)	
*Wealth index*		0.07		0.70
Poorest	1		1	
Poorer	1.35 (0.78–2.33)		1.35 (0.76–2.40)	
Middle	1.64 (0.93–2.89)		1.57 (0.85–2.89)	
Richer	1.61 (0.93–2.79)		1.38 (0.75–2.52)	
Richest	2.31 (1.29–4.15)		1.37 (0.66–2.84)	
*Owns mobile phone*		**0.01**		0.11
No	1		1	
Yes	1.57 (1.12–2.20)		1.32 (0.94–1.85)	
*Region*		0.12		0.30
Kigali	1		1	
South	0.55 (0.31–1.00)		0.63 (0.35–1.13)	
West	0.79 (0.43–1.45)		0.82 (0.44–1.53)	
North	0.47 (0.24–0.91)		0.52 (0.27–1.01)	
East	0.64 (0.35–1.19)		0.72 (0.39–1.34)	
*Residence*		**0.04**		0.98
Rural	1		1	
Urban	1.56 (1.01–2.40)		1.01 (0.57–1.79)	

* CI* confidence interval,* RDHS* Rwanda demographic health survey,* HIV/AIDS* Human immunodeficiency virus/Acquired immune deficiency syndrome

Bold = significant, *significant at 0.25, **significant at 0.05

Health insurance coverage was also identified as a significant predictor of comprehensive HIV/AIDS knowledge. Men with health insurance exhibited nearly twice the odds of possessing accurate HIV knowledge compared to their uninsured counterparts. This may reflect increased engagement with the healthcare system among insured individuals, facilitating greater access to health education, counseling, and HIV-related information. Similar associations have been reported in other contexts, where improved access to healthcare services correlates with higher levels of disease awareness and preventive behavior [33, 36]. Thus, policies aimed at expanding health insurance coverage - particularly among older adults - could play a pivotal role in enhancing HIV knowledge and reducing disease burden on this demographic.

Other factors, such as internet usage and mobile phone ownership, demonstrated associations with comprehensive HIV/AIDS knowledge in the bivariable analysis but lost significance after adjustment in the multivariable model. This suggests that these variables may exert indirect effects through more proximal determinants, such as education and healthcare access. Nevertheless, digital and media-based health communication strategies remain essential components of public health outreach. Efforts to harness these platforms to disseminate accurate, age-appropriate HIV information could further strengthen knowledge among older adults, particularly in hard-to-reach or underserved communities.

This study has several limitations that should be considered when interpreting the findings. First, comprehensive HIV knowledge was measured as a binary variable derived from six standardized DHS questions, which may not fully capture the depth or multidimensional nature of participants’ understanding. Although this approach follows DHS convention, a sensitivity analysis using alternative scoring criteria (≥ 5/6 and a continuous 0–6 scale) indicated consistent patterns, enhancing confidence in the results. Second, because the DHS men’s survey includes respondents aged 15–59 years, men aged 60 and above were excluded, limiting the generalizability of the findings to the oldest age groups. Third, all data were self-reported, which may be subject to recall and social desirability biases, particularly for sensitive topics such as HIV/AIDS. Fourth, the cross-sectional design precludes causal inference between the identified predictors and comprehensive HIV knowledge. Finally, while the model incorporated predisposing, enabling, and contextual factors guided by the conceptual framework, unmeasured variables-such as cultural norms or prior HIV testing experiences-may also influence knowledge levels. Despite these limitations, this study provides valuable insights as one of the first analyses of HIV knowledge among Rwandan men aged 50–59 years.

## Conclusions

This study offers critical insights into the level and determinants of comprehensive HIV/AIDS knowledge among Rwandan men aged 50–59 a demographic frequently underrepresented in HIV prevention research. Although nearly 70% of older men demonstrated comprehensive knowledge, this remains below global targets, underscoring the need for strengthened educational and public health initiatives. Secondary education and health insurance coverage were identified as the strongest independent predictors of HIV knowledge in this population. While cross-sectional and descriptive, these results can inform where to situate age-tailored information-e.g., brief counseling at insurance renewal points and radio/CHW messaging channels frequented by men in their fifties.

## Data Availability

All data underlying the findings described in this manuscript are fully available without restriction. The dataset used for this analysis can be accessed through the Demographic and Health Surveys (DHS) Program website at (https://dhsprogram.com/). Processed data supporting the results are presented within the paper.

## Abbreviations

AIDS: Acquired Immune Deficiency Syndrome
AOR: Adjusted Odds Ratio
AR: T Antiretroviral Therapy
CI: Confidence Interval
COR: Crude Odds Ratio
DHS: Demographic and Health Survey
HIV: Human Immunodeficiency Virus
PMTCT: Prevention of Mother-to-Child Transmission
PNLS: Programme National de Lutte contre le SIDA (National Program for HIV/AIDS Control)
RDHS: Rwanda Demographic and Health Survey
SPSS: Statistical Package for Social Science
UNAIDS: Joint United Nations Programme on HIV/AIDS
VIF: Variance Inflation Factor
VMMC: Voluntary Medical Male Circumcision
WHO: World Health Organization
